# Automated diagnosing primary open-angle glaucoma from fundus image by simulating human’s grading with deep learning

**DOI:** 10.1038/s41598-022-17753-4

**Published:** 2022-08-18

**Authors:** Mingquan Lin, Bojian Hou, Lei Liu, Mae Gordon, Michael Kass, Fei Wang, Sarah H. Van Tassel, Yifan Peng

**Affiliations:** 1 Department of Population Health Sciences, Weill Cornell Medicine, New York, NY USA; 2 Institute for Public Health, Washington University School of Medicine, St. Louis, MO USA; 3 Department of Ophthalmology and Visual Sciences, Washington University School of Medicine, St. Louis, MO USA; 4 Department of Ophthalmology, Weill Cornell Medicine, New York, NY USA

**Keywords:** Eye diseases, Diagnosis

## Abstract

Primary open-angle glaucoma (POAG) is a leading cause of irreversible blindness worldwide. Although deep learning methods have been proposed to diagnose POAG, it remains challenging to develop a robust and explainable algorithm to automatically facilitate the downstream diagnostic tasks. In this study, we present an automated classification algorithm, GlaucomaNet, to identify POAG using variable fundus photographs from different populations and settings. GlaucomaNet consists of two convolutional neural networks to simulate the human grading process: learning the discriminative features and fusing the features for grading. We evaluated GlaucomaNet on two datasets: Ocular Hypertension Treatment Study (OHTS) participants and the Large-scale Attention-based Glaucoma (LAG) dataset. GlaucomaNet achieved the highest AUC of 0.904 and 0.997 for POAG diagnosis on OHTS and LAG datasets. An ensemble of network architectures further improved diagnostic accuracy. By simulating the human grading process, GlaucomaNet demonstrated high accuracy with increased transparency in POAG diagnosis (comprehensiveness scores of 97% and 36%). These methods also address two well-known challenges in the field: the need for increased image data diversity and relying heavily on perimetry for POAG diagnosis. These results highlight the potential of deep learning to assist and enhance clinical POAG diagnosis. GlaucomaNet is publicly available on https://github.com/bionlplab/GlaucomaNet.

## Introduction

Primary open-angle glaucoma (POAG) is one of the leading causes of blindness in the US and worldwide^1^. It has been projected to affect approximately 111.8 million people by 2040. Among these patients, 5.3 million may be bilaterally blind^2^. In the United States, POAG is the most common form of glaucoma and is the leading cause of blindness among African-Americans^3^ and Hispanics^4^. POAG is asymptomatic until it reaches an advanced stage when visual field (VF) loss occurs. Therefore, accurately identifying individuals with glaucoma is critical to clinical decision-making, which can help inform the need for medical and surgical treatments and patient monitoring^5,6^.

Screening is an efficient and effective way to detect POAG to prevent blindness^7^. However, screening needs expensive facilities and experienced ophthalmologists, which causes low prevalence^7^. Meanwhile, fundus photography is convenient and inexpensive for recording optic nerve head structure. But detecing POAG on fundus photographs still requires human labor and severely depends on experienced ophthalmologists. Therefore, it is important to develop an automatic model to detect POAG with high accuracy and low cost from fundus photographs.

Developments in artificial intelligence have provided potential opportunities for automatic POAG diagnosis using fundus photographs. Singh et al. segmented optic disc and optic cup images to obtain the vertical cup-to-disc ratio (VCDR) and extracted the handcrafted features from the VCDR to perform the classification^8^. Acharya et al. extracted texture features and higher-order spectral features to detect POAG based on a Support Vector Machine (SVM) and Naïve Bayesian (NB) classifier^9^. Dua et al. also used SVM and NB to classify the wavelet-based energy features^10^. An adaptive threshold-based image processing method was adopted by Issac et al. for POAG classification^11^. However, these methods are influenced by the segmentation accuracy of the optic disc and optic cup or have relatively low classification accuracy because they only consider handcrafted features. Recently, deep learning methods have demonstrated promising results in biology and medicine^12–27^. For POAG detection, several works directly used Convolutional Neural Networks (CNN) to detect glaucoma^28–33^. Fu et al. provided a novel dis-aware ensemble network for glaucoma screening from fundus images^34^. Li et al. put forward a framework that integrates holistic and local deep features for glaucoma classification^35^.

While these studies have brought significant improvements, several limitations hinder their translation into clinical practice. First, most models were developed on small datasets from a single institution^28,29,34,35^. It makes the methods less generalizable to different populations and settings. Second is the requirement of refined image annotation such as attention maps^30^, image cropping^32^, and optic nerve head segmentation^33^. Today, it remains challenging to build highly-accurate systems using image-level labels. Finally, all these works lack interpretability which reduces their utility for medical applications. Thus, it is critical to develop an interpretable AI system for ophthalmologists besides providing a fast and accurate POAG diagnosis. Specifically, interest has recently grown in designing clinical systems that can reveal why models make specific predictions^36–38^. To the best of our knowledge, few works in POAG diagnosis have been studied in this direction.

This work addresses these issues by proposing a joint fusion network (GlaucomaNet) to accurately and robustly diagnose POAG using variable fundus photographs. Different from previous studies, GlaucomaNet consists of two convolutional neural networks to simulate the human grading process: one performing preliminary grading and the other detailed grading. It is trained in an end-to-end strategy without manual cropping so that the whole training process is fast and less labor-intensive and can be quickly deployed to provide a first-line assessment. In addition, an ensemble of network architectures further improved diagnostic accuracy. We validated the models on two independent datasets (Table 1). One is the Ocular Hypertension Treatment Study (OHTS) cohort, one of the largest longitudinal clinical trials in POAG (1636 participants and 37,399 images) from 22 centers in the United States^39^. The other is the Large-scale Attention-based Glaucoma (LAG) database, a publicly available database collected at the Chinese Glaucoma Study Alliance and Beijing Tongren Hospital^30^. GlaucomaNet achieved the highest AUC for POAG diagnosis on these two datasets. To further evaluate the rationale of our model, we consider the prediction from the model for the POAG diagnosis once the supporting rationales (optic disc) are stripped. To this end, we constructed a new dataset by masking the optic disc portion of the fundus photographs. When stripping the bounding box of optic discs and random bounding boxes were masked, we showed that the rationales (optic disc) are influential in the POAG diagnosis in our network.

**Table 1:** Characteristics of the OHTS and LAG datasets.

Dataset	OHTS	LAG
#Participants	1636	4855
#Images	37,339	4915
#POAG	2321	1711
#Normal	35,018	3144

## Methods

### Data acquisition

In this study, we include two independent datasets. These two databases are large, cross-sectional, longitudinal, and population-based studies. All participants provided informed consent at original study entry. This study does not need institutional review board approval because it does not constitute human subjects research. All experiments were performed in accordance with relevant guidelines and regulations.

#### Ocular hypertension treatment study

The first dataset was obtained from the Ocular Hypertension Treatment Study (OHTS)^39^. It is one of the largest longitudinal clinical trials in POAG from 22 centers in the United States. The study protocol was approved by an independent Institutional Review Board at each clinical center.

The participants in this dataset were selected according to both eligibility and exclusion criteria^39^. Briefly, the eligibility criteria include intraocular pressure (IOP) (between 24 and 32 mmHg in one eye and between 21 and 32 mmHg in the fellow eye) and age (between 40 and 80 years old). The visual field tests were interpreted by the Visual Field Reading Center, and stereoscopic photographs of the optics discs were interpreted by the Optic Disc Reading Center. Exclusion criteria included previous intraocular surgery, visual acuity worse than 20/40 in either eye, and diseases that may cause optic disc deterioration and visual field loss (such as diabetic retinopathy).

The reading center workflow has been described in detail in Kass et al.^39^. In brief, two certified readers independently assessed optic disc deterioration. If there was disagreement between two readers, a senior reader adjudicated in a masked fashion. The POAG diagnosis in a quality control sample of 86 eyes (50 normal eyes and 36 with progression) showed test–retest agreement at κ = 0.70 (95% confidence interval [CI], 0.55–0.85).

**Figure 1:**
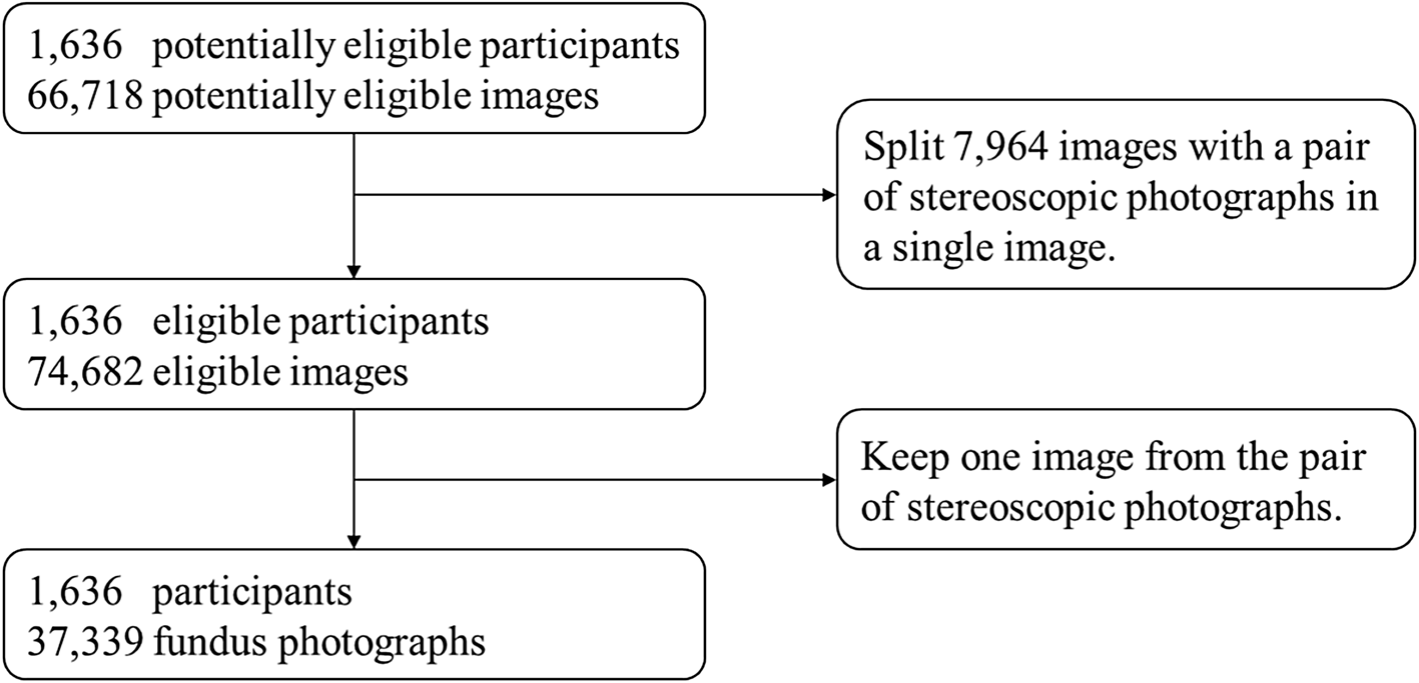
Creation of the OHTS dataset.

Figure 1 shows the creation of the OHTS dataset in our study. Because of the inherent redundancy in a pair of stereoscopic photographs, we used only one of the photographs for each eye. There are 7964 images (11.9%) that contain the stereo pair in a single image file. We split them into two images. In the end, we obtained 39,339 fundus photographs from 3272 eyes of 1636 subjects. On the image level, 6.2% (2321) of fundus photographs were diagnosed with POAG (Table 1).

#### Large-scale Attention-based Glaucoma database

The second dataset was obtained from the Large-scale Attention-based Glaucoma (LAG) database, a publicly available database collected at the Chinese Glaucoma Study Alliance and Beijing Tongren Hospital^40^ (https://github.com/smilell/AG-CNN). LAG contains 4855 fundus images, of which 35% (1711) have POAG. This dataset has a high proportion of glaucoma cases (41.2%), exceeding what would be expected when screening for glaucoma, which was designed intentionally to create a balanced sample. All research adhered to the tenets of the Declaration of Helsinki. Qualified glaucoma specialists diagnosed each fundus image by considering both morphologic and functional analyses such as intraocular pressure, visual field loss, and manual optic disc assessment. Finally, all fundus images were confirmed with positive or negative glaucoma, seen as the gold standard.

### Model development

#### Overall architecture

**Figure 2:**
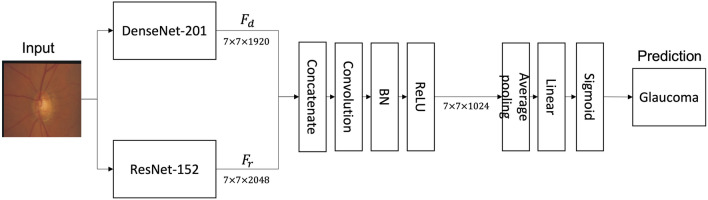
The architecture of the proposed GlaucomaNet.

GlaucomaNet comprises two convolutional blocks followed by seven layers (Fig. 2). First, a single fundus image ${x}_{i}$ is passed through two convolutional neural networks, DenseNet-201^41^ and ResNet-152^42^. For DenseNet-201, we used the output of the 200 layers. For ResNet-152, we used the output of the 151st layer. We denote the outputs as ${F}_{d}$ and ${F}_{r}$, respectively. We then concatenated ${F}_{d}$ and ${F}_{r}$ and used $1\times 1$ convolution (conv1d) followed by a batch normalization layer (BN)^43^ and rectified linear units (ReLU)^44^ as transition layers. In the end, a global average pooling (AvgPooling)^45^ with a fully connected layer is performed, and a sigmoid classifier is attached.

$${F}_{d}=DenseNet 201({x}_{i})$$

$${F}_{r}=ResNet152({x}_{i})$$

$${F}_{1}=conv1d\left(concat\left({F}_{d}, {F}_{r}\right)\right)$$

$${F}_{2}=ReLU\left(BN\left({F}_{1}\right)\right)$$

$${F}_{3}=AvgPooling({F}_{2})$$

$$o={sigmoid(W}_{3}{F}_{3}+{b}_{3})$$

#### Loss function

On both datasets, there is a severe class imbalance between non-POAG and POAG images. For example, only 6.22% of images in the OHTS dataset contain POAG. To overcome this data imbalance issue, we applied weighted cross-entropy^46^, a commonly used loss function in classification. The adopted weighted cross-entropy was as follows:

$$\mathcal{L}=-\frac{1}{N}\sum_{n=1}^{N}[{w}_{1}{y}_{n}\mathit{log}\left({\widehat{y}}_{n}\left({x}_{n},{\theta }_{s}\right)\right)+{w}_{2}(1-{y}_{n})\mathit{log}\left(1-{\widehat{y}}_{n}\left({x}_{n},{\theta }_{s}\right)\right)]$$

where $N$ is the number of training examples, ${w}_{1}$ and ${w}_{2}$ are controlling hyperparameters, ${y}_{n}$ is the ground truth while ${\widehat{y}}_{n}$ is the likelihood predicted by the classifier, and ${\theta }_{s}$ represents the parameters of the neural network.

#### Image augmentation

A stochastic image augmentation was randomly applied to transform a fundus photograph into an augmented view. In this work, we sequentially appled three simple augmentation operations: (1) random rotation between 0° and 10°, (2) random translation: an image was translated randomly along the x- and y-axes by distances ranging from 0 to 10% of width or height of the image, and (3) random flipping. These data augmentation operations are crucial in increasing the diversity of the dataset and thus yielding effective and robust representations^24^.

#### Training strategy

We first trained two different single models, DenseNet-201^41^ and ResNet-152^42^. Then, we selected the convolutional part of the DenseNet-201and ResNet-152 as the convolutional part of the proposed model. Afterward, we trained the entire network in an end-to-end manner. During the training, we updated the entire network parameters. Therefore, the loss was propagated back to the individual neural networks, creating better feature representations for each training iteration. Since we learned the feature representations from intermediate layers, our training strategy is closed to a joint fusion strategy^47^.

To use the pre-trained model on ImageNet, all images were resized to 224 × 224. All models were implemented by Keras with a backend of Tensorflow. The proposed network was optimized using the Adam optimizer method^48^. The learning rate was $5\times {10}^{-5}$. The experiments were performed on Intel Core i9-9960 X 16 cores processor and NVIDIA Quadro RTX 6000 GPU.

#### Ensemble methods

In machine learning, ensemble methods combine multiple models to produce improved results, surpassing single models. This study also evaluated the performance of three ensemble methods: macro averaging, random forest^49^, and linear regression. The macro averaging model averages the predicted class probabilities of the seven models together. The random forest model computes a number of decision trees. The final classification corresponds to the majority vote among the individual trees. Here, we used the predicted class probabilities of the seven models and trained the random forest using 500 trees on the development set. For linear regression, we also used the predicted class probabilities of the seven models as input.

#### Model’s clinical rationales

State-of-the-art models for POAG diagnosis, including ours, are now predominantly deep neural networks that are opaque in terms of how they come to make predictions. This limitation has increased interest in designing more interpretable deep models that reveal the ‘reasoning’ behind model outputs^50^. One common way to explain the deep learning model is through a saliency map where important regions on the images are highlighted to correspond to the disease of interest^22,24,32^. For POAG diagnosis, segmented optic disc and optic cup are typically used. However, such spatial annotation requires substantial effort and is not generally available in large quantities. In addition, such approaches do not establish whether the model relied on these regions to make a prediction.

**Figure 3:**
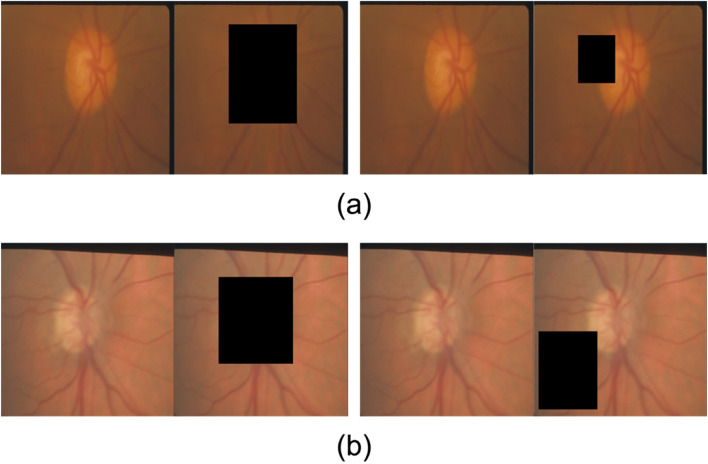
Examples of fundus photographs and their corresponding masked images: (**a**) POAG due to VF only (the left one is based on the optic disc mask and the right one is based on a random mask) and (**b**) POAG due to glaucomatous disc criteria (the left one is based on the optic disc mask and the right one is based on a random mask).

In this work, we proposed a different way to assess the plausibility of rationales by measuring rationale faithfulness—rationales ought to have meaningfully influenced its prediction^51,52^. Following DeYoung et al.^53^, we conducted a contrasting experiment to explore the rationales of the optic disc. Figure 3 provides two demos for the experiment.

First, we manually masked the optic disc ${r}_{i}$ from the fundus photograph with a bounding box. Let $m\left({x}_{i}\right)$ be the original prediction provided by a model $m$. We then consider the predicted class from the model once the supporting bounding box is stripped. Intuitively, the model should generate a less “correct” class. We then measure this as comprehensiveness

$$comprehensiveness=\frac{1}{n}{\sum }_{n}(m\left({x}_{i}\right)-m\left({x}_{i}/{r}_{i}\right)).$$

In particular, if we selected glaucomatous images where their labels were predicted by the model correctly ($m\left({x}_{i}\right)\equiv 1$), a score of 1.0 indicates that the optic discs (rationale) are indeed influential in the prediction, while 0.0 indicates that the rationales are not the reasons for the prediction.

For comparison, we also randomly masked the image without completely overlapping the optic disc’s bounding box. The overlapping ratio (i.e., the intersection of the union (IOU)) is no larger than a threshold. Specifically, we randomly selected the IOU’s threshold from 1/2, 2/5, 1/3, and 1/4. The ratio of the area of the original bounding box can also be variant (1, 1/4, 1/9, and 1/16). Finally, we generated ten images with bounding boxes of different thresholds and areas.

### Evaluation metrics

Our experiments report accuracy, precision, sensitivity (recall), specificity, and F1-score.

$$Accuracy=\frac{TP+TN}{TP+TN+FP+FN}, Precision=\frac{TP}{TP+FP}, Sensitivity= \frac{TP}{TP+FN}, Specificity= \frac{TN}{TN+FP}, F1-score=2\frac{precision\bullet recall}{precision+recall}$$

TP, TN, FP, and FP denote true positive, true negative, false positive, and false negative. In addition, we report the AUC (Area Under the Curve) ROC (Receiver Operating Characteristics) curve. A ROC curve plots true positive rate (TPR, also called sensitivity) vs. false positive rate (FPR) at different classification thresholds. These metrics were assessed by the Mann–Whitney U test. The Mann–Whitney U test is associated with a p-value, which is the probability of obtaining a mean difference between two methods as extreme as observed in the test, assuming the null hypothesis is correct (i.e., there is no difference between the results generated by two models).

In this study, we used the fivefold cross-validation to obtain a distribution of the experimental metrics and reported standard deviations. For the OHTS dataset, we split the entire dataset randomly into five groups at the patient level. This ensured that no participant was in more than one group to avoid cross-contamination between the training and test datasets. In each fold of the cross-validation, we took one group (20% of total subjects) as the hold-out test set and the remaining 4 groups as the training set. For the LAG dataset, we conducted fivefold cross-validation using the same strategy.

## Results

### POAG diagnosis on the OHTS dataset

**Figure 4:**
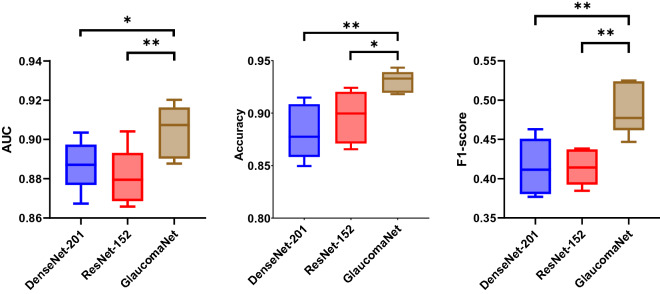
Comparison of different metrics (standard deviation) for different model architectures in the OHTS dataset. p-values are calculated between GlaucomaNet and other models. *p-value ≤ 0.05, **p-value ≤ 0.01.

The performance was compared with DenseNet-201 and ResNet-152 (Fig. 4) and four state-of-the-art neural networks, VGG-16^54^, NASNetMobile^55^, Xception^56^, MobileV2^57^, and ResNet-50^42^ (Supplementary Table S1). Our model achieved the best results, with an accuracy of 0.930, an F1-score of 0.490, and an AUC of 0.904. Compared to the best baseline (DenseNet-201), GlaucomaNet has higher accuracy (4.80%), F1-score (7.50%), and AUC (1.70%). The p-value indicates that accuracy, precision, specificity, F1-score, and AUC obtained by GlaucomaNet are statically higher than state-of-the-art neural networks. From Supplementary Table S1, we also found that DenseNet-201 and ResNet-152 achieved better results than other baselines (1.7–14.9%). It is sufficient to compare our model to these two models hereafter.

**Figure 5:**
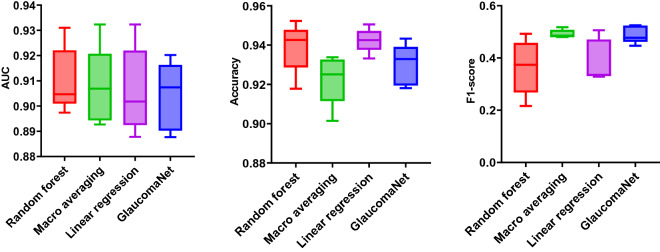
Comparison of different metrics (standard deviation) for proposed and ensemble methods in the OHTS dataset.

By combining the different network architectures in a model ensemble, we found that the random forest method improved accuracy to 0.939 and AUC to 0.910 (Fig. 5 and Supplementary Table S2). Meanwhile, the F1-score decreased, leading to reduced sensitivity. However, these changes were not statistically significant.

To evaluate the model’s rationales, we randomly selected 100 “correctly predicted” glaucomatous fundus photographs from the OHTS dataset. After stripping the bounding box of optic discs, 97% of images (comprehensiveness score of 97%) were detected as “normal” by GlaucomaNet (Supplementary Table S3). On the contrary, 36% of images (comprehensiveness score of 36%) were detected as “normal” when the random bounding boxes were masked. The results demonstrate that our model is dramatically less confident in its POAG prediction once the optic discs are masked. We applied DenseNet-201 to the same experimental settings and obtained comprehensiveness scores of 82% and 20%, respectively. The comparison between GlaucomaNet and DenseNet-201 suggests that optic discs are more needed in our model to predict POAG from fundus photographs—that is, our model is more rational than DenseNet.

Supplementary Figure S1 presents more specific results by histograms. The scores for the IOU’s thresholds 1/2, 2/5, 1/3, and 1/4 are 40.3%, 38.4%, 33.0%, and 30.7%, and 25.4%, 23.6%, 18.0%, and 12.9% for GlaucomaNet and DenseNet-201, respectively; the scores for the area ratios 1, 1/4, 1/9, and 1/16 are 65.7%, 32.8%, 26.4%, and 21.1%, and 47.0%, 13.4%, 11.7%, and 8.8% for GlaucomaNet and DenseNet-201, respectively.

### POAG diagnosis on the LAG dataset

**Figure 6:**
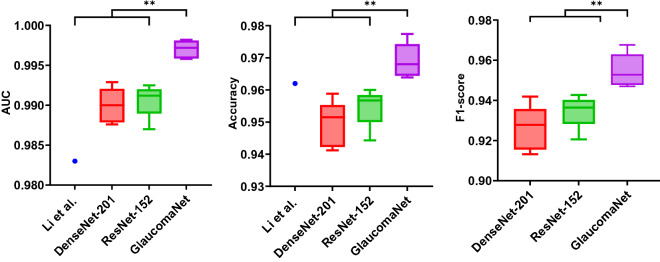
Comparison of different metrics for different model architectures in the LAG dataset. The model reported in Li et al. was trained on 10,928 images, with 4528 having POAG. There is no reported F1-score for Li et al. P-values are calculated between GlaucomaNet and other models. *p-value ≤ 0.05, **p-value ≤ 0.01.

Figure 6 compares the results of GlaucomaNet with DenseNet-201 and ResNet-152 on the LAG dataset. Our model obtained the best results, with an accuracy of 0.969, an F1-score of 0.955, and an AUC of 0.997 (Supplementary Table S4). GlaucomaNet also outperforms the state-of-the-art methods for all evaluation metrics as shown in Supplementary Table S4. In addition, we include the results reported in Li et al.^30^, although they used a much larger LAG dataset to train the model. GlaucomaNet achieves superior results even though we only used 41.3% of the LAG dataset for training.

### T-distributed stochastic neighbor embedding (t-SNE) method

**Figure 7:**
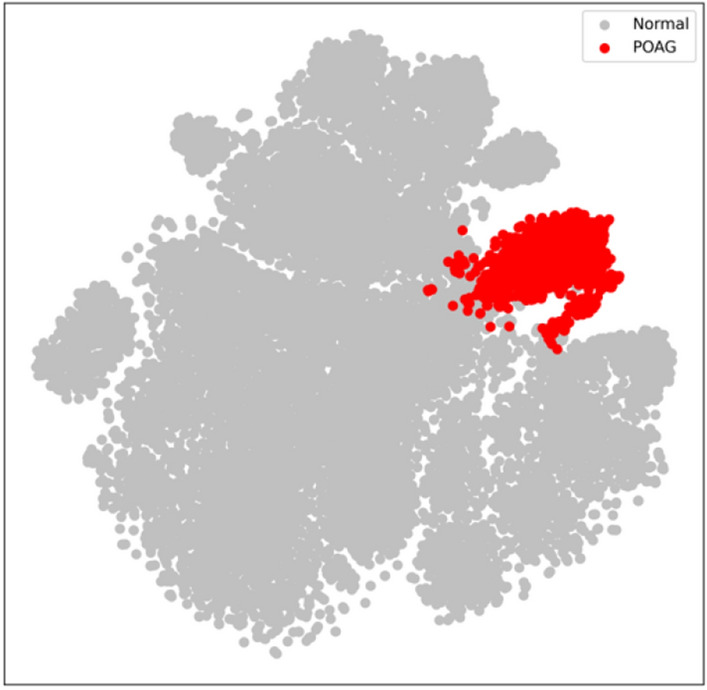
The t-SNE visualization of the proposed model on the OHTS dataset. Each point represents a fundus image. Red and gray dots represent the glaucomatous and non-glaucomatous images.

In this study, the internal features learned by GlaucomaNet were studied using t-distributed Stochastic Neighbor Embedding, which is well suited for visualizing high-dimensional datasets^58^. We first obtained the 1024-dimensional features from the output of averaging pooling layer based on the proposed model. Then, we applied the t-SNE technique to reduce the vector into 2 dimensions for visualization. Figure 7 demonstrates that glaucomatous images can be separated from non-glaucomatous images.

## Discussion

The primary aim of this study is to develop a fully automatic deep learning network for POAG diagnosis using fundus photographs. On two datasets, our proposed model was superior to the state-of-the-art model. Unlike single neural networks such as DenseNet, our method fused the features from two state-of-the-art networks. This simulates the fusion of two readers who first learn the discriminative features separately and then fuse the features for grading.

Our experiment also shows that ensemble methods produce more accurate solutions than a single model. On the OHTS dataset, the random forest achieves the highest AUC. There might be two reasons behind this. First, each tree in the random forests is built from a sample drawn with a replacement from the training set. Second, random forests select a subset of features, rather than all features to train the model. As can be seen from Supplementary Table S2, its variance decreases, resulting in an overall better model.

Of note, although most evaluation metrics of our proposed model were superior, the sensitivity was lower than other models on the OHTS dataset. One potential reason is that the positive (glaucomatous) examples for model training in the OHTS dataset were as low as 11.9% of the total images. We postulate that further training our model with a larger number of POAG images may improve its performance. We also noticed two studies conducted on OHTS. One was performed by Thakur et al.^32^, which achieved an AUC of 0.945. There are two main differences between our approach and theirs. First, we used the whole OHTS dataset while Thakur et al.^32^ discarded 24% of the fundus photographs with poor image quality. We believe our experimental settings allow us to more closely access the model’s real-world clinical performance. Second, we did not manually crop the images in the data preprocessing stage, which minimizes the labor required to deploy our model to healthcare centers with a quick turn-around time and again more closely resembles a real-world workflow. To make a fair comparison, we applied the same method in Thakur et al.^32^ to our dataset (MobileV2 in Supplementary Table S1). The AUC was 0.799, lower than the GlaucomaNet under the same setting. The other study was performed by Fan et al.^59^, which achieved an AUC of 0.88 based on optic disc or VF change attributable to POAG by the Endpoint Committee (the same criteria as us). We applied their model (ResNet-50) to our data split under the same setting and achieved the AUC of 0.863, which is similar to the result obtained by Fan et al. Because we used different data split and preprocessing methods, the results are not strictly comparable.

We also observed that the performance of GlaucomaNet varies dramatically across two datasets. There are potentially three reasons. First, digital fundus photographs have a higher resolution than digitized film fundus photographs. The LAG dataset contains digital fundus photographs, while OHTS contains digitized film fundus photographs. However, GlaucomaNet can still get an AUC of 0.904 on the OHTS dataset. Second, the images in the LAG datasets were carefully selected by the ophthalmologists, and all glaucomatous images are due to structural glaucomatous optic nerve abnormalities. In contrast, some of POAG fundus photographs in OHTS are due to visual field changes in the absence of glaucomatous disc changes over time. Therefore, the task on LAG is less challenging than OHTS.

### Limitation and future work

One limitation of our proposed model arises from the imbalance in the dataset used for its training, particularly the extremely low proportion of images with POAG. As described previously, this likely contributed to the relatively lower sensitivity. However, as evidenced by the results on more balanced LAG, this limitation may be addressed by further training using image datasets with a higher proportion of POAG cases.

Another limitation lies in detecting POAG eyes due to visual field abnormality without any obvious structural sign of Glaucomatous Optic Neuropathy (GON). These images had a higher tendency to be missed by our model. It would be interesting for future studies to differentiate POAG due to VF abnormality and GON.

In conclusion, this study proposed a new end-to-end deep learning network for automatic POAG detection from fundus photographs. Two datasets were used to evaluate the proposed model. The results demonstrated that the proposed network has a good performance on POAG diagnosis. We also analyzed the performance of several ensemble methods; lessons from these approaches may have applicability to developing deep learning models for other retinal diseases, such as diabetic retinopathy, age-related macular degeneration, and even for image-based deep learning systems outside of ophthalmology. Although deep learning models are often considered “black-box” entities, we aimed to improve the transparency of our algorithm by constructing a new dataset by masking the optic disc on the fundus photographs. These “contrasting” examples help us understand if the rationales (optic disc) influence the POAG diagnosis. These efforts to demystify deep learning models may help improve levels of acceptability to patients and adoption by ophthalmologists.

The deep learning model and data partition are publicly available (https://github.com/bionlplab/‌GlaucomaNet). By making them available, we aim to maximize this study's transparency and reproducibility and provide a benchmark for further refinement and development of the algorithm. In addition, this deep learning model, trained on one of the largest POAG fundus photograph datasets, may allow for future deep learning studies on POAG diagnosis where only smaller datasets are available.

In the future, we aim to improve the model by incorporating multi-modal data. It would also be interesting to compare the model's accuracy with different groups of ophthalmologists (e.g., graders at Reading Centers, glaucoma specialists, general ophthalmologists, and trainee ophthalmologists). These results could then be tested and validated by further studies from different countries and populations. In that case, it is possible that the integration of deep learning models into clinical practice might become increasingly acceptable to patients and ophthalmologists and may ultimately enhance clinical decision-making.

## Supplementary Information

Supplementary Figure S1.

Supplementary Tables.

## Data Availability

The Ocular Hypertension Treatment Study (OHTS) dataset is available upon request due to patient protection (https://ohts.wustl.edu/), please contact the author in^39^ (Michael Kas: kass@wustl.edu). The large-scale attention-based glaucoma (LAG) database is available in https://github.com/smilell/AG-CNN (please contact liliu1995@buaa.edu.cn or liliu419@foxmail.com to get the password to download the dataset).
