# Supplementary material for: Automated diagnosing primary open-angle glaucoma from fundus image by simulating human’s grading with deep learning
Source: Sci Rep. 2022 Aug 18;12:14080. doi: 10.1038/s41598-022-17753-4 (PMC9388536; doi:10.1038/s41598-022-17753-4)
Supplement: Supplementary file 1 — Supplementary Figure S1. [file 41598_2022_17753_MOESM1_ESM.docx]

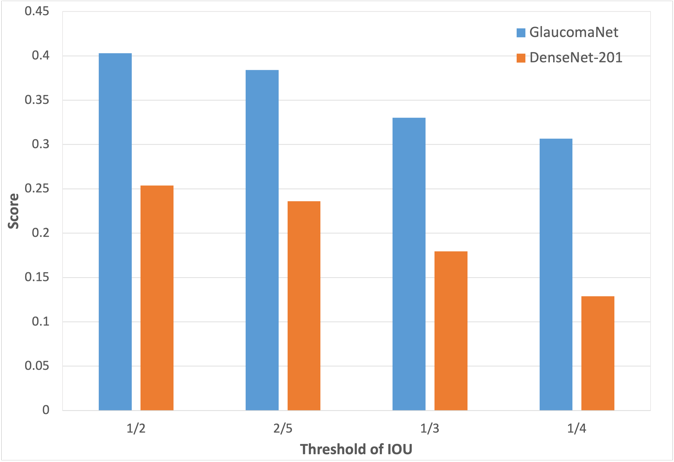

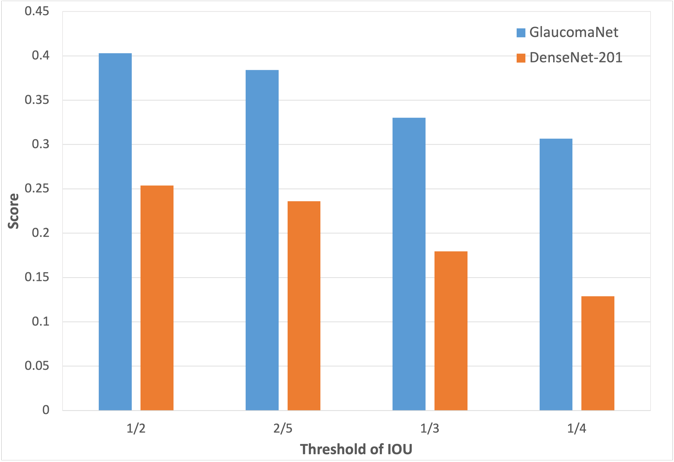


1. (b)

Figure S1. The comprehensiveness score with respect to (a) the ratio of the area of the optic disc bounding box; (b) the threshold of IOU.
