# Supplementary material for: Automated diagnosing primary open-angle glaucoma from fundus image by simulating human’s grading with deep learning
Source: Sci Rep. 2022 Aug 18;12:14080. doi: 10.1038/s41598-022-17753-4 (PMC9388536; doi:10.1038/s41598-022-17753-4)
Supplement: Supplementary file 2 — Supplementary Tables. [file 41598_2022_17753_MOESM2_ESM.docx]

Table S1 Comparison of different metrics (standard deviation) for different model architectures in the OHTS dataset. ­*­*p-values are calculated between GlaucomaNet and other models. *: p-value ≤ 0.05, **: p-value ≤ 0.01.

| Method | Accuracy | Precision | Sensitivity | Specificity | F1-score | AUC |
| --- | --- | --- | --- | --- | --- | --- |
| DenseNet-201 | 0.882 (0.026)^**^ | 0.309 (0.053)^**^ | **0.660 (0.082)**^*^ | 0.897 (0.033)^**^ | 0.415 (0.036)^**^ | 0.887 (0.013)^*^ |
| ResNet-152 | 0.896 (0.025)^*^ | 0.325 (0.062)^**^ | 0.586 (0.120) | 0.917 (0.035)^*^ | 0.415 (0.023)^**^ | 0.881 (0.015)* |
| VGG-16 | 0.884 (0.019)^**^ | 0.290 (0.049)^**^ | 0.583 (0.111) | 0.904 (0.018)^*^ | 0.386 (0.060)^**^ | 0.865 (0.044)* |
| NASNetMobile | 0.797 (0.083)^**^ | 0.170 (0.068)^**^ | 0.479 (0.115) | 0.818 (0.094)^*^ | 0.240 (0.061)^**^ | 0.732 (0.066)^**^ |
| Xception | 0.877 (0.015)^**^ | 0.273 (0.021)^**^ | 0.576 (0.071) | 0.897 (0.019)^**^ | 0.369 (0.022)^**^ | 0.844 (0.030)^**^ |
| MobileV2 | 0.753 (0.116)^**^ | 0.163 (0.042)^**^ | 0.637 (0.233) | 0.761 (0.137)^**^ | 0.250 (0.054)^**^ | 0.799 (0.056)^**^ |
| ResNet-50 | 0.819 (0.060)^**^ | 0.224 (0.043)^**^ | 0.704 (0.111)^*^ | 0.826 (0.070)^**^ | 0.334 (0.038)^**^ | 0.863 (0.016)^*^ |
| GlaucomaNet | **0.930 (0.010)** | **0.457 (0.055)** | 0.538 (0.067) | **0.956 (0.014)** | **0.490 (0.034)** | **0.904 (0.014)** |

Table S2. Comparison of different metrics (standard deviation) for proposed and ensemble methods in the OHTS dataset. ­*­*p-values are calculated between GlaucomaNet and other models. *: p-value ≤ 0.05, **: p-value ≤ 0.01.

| Method | Accuracy | Precision | Sensitivity | Specificity | F1-score | AUC |
| --- | --- | --- | --- | --- | --- | --- |
| Random forest | 0.939 (0.013) | **0.620 (0.148)** | 0.310 (0.196) | **0.981 (0.025)** | 0.365 (0.105) | **0.910 (0.013)** |
| Macro averaging | 0.922 (0.014) | 0.422 (0.042) | **0.597 (0.098)** | 0.943 (0.021) | 0.489 (0.021) | 0.905 (0.016) |
| Linear regression | **0.942 (0.006)** | 0.614 (0.094)^*^ | 0.309 (0.141)^*^ | 0.979 (0.016) | 0.388 (0.080) | 0.906 (0.017) |
| GlaucomaNet | 0.930 (0.010) | 0.457 (0.055) | 0.538 (0.067) | 0.956 (0.014) | **0.490 (0.034)** | 0.904 (0.014) |

Table S3. The comprehensiveness score of DenseNet-20 and GlaucomaNet for 100 images with optic discs masked, and 1,000 images with non-optic discs masked.

| Method | Optic discs masked | Non-optic discs masked |
| --- | --- | --- |
| DenseNet-201 | 82% | 20% |
| GlaucomaNet | 97% | 36% |

Table S4. Comparison of different metrics for different model architectures in the LAG dataset. ­­p-values are calculated between GlaucomaNet and other models. *: p-value ≤ 0.05, **: p-value ≤ 0.01.

| Method | Accuracy | Precision | Sensitivity | Specificity | F1-score | AUC |
| --- | --- | --- | --- | --- | --- | --- |
| Li et al. ^†^ | 0.962 | 0.954 | - | 0.967 | - | 0.983 |
| DenseNet-201 | 0.949 (0.007)^**^ | 0.952 (0.016)^**^ | 0.902 (0.028) | 0.976 (0.010)^*^ | 0.926 (0.011)^**^ | 0.990 (0.002)^**^ |
| ResNet-152 | 0.955 (0.006)^**^ | 0.951 (0.016)^**^ | 0.919 (0.012) | 0.975 (0.009)^**^ | 0.935 (0.008)^**^ | 0.990 (0.002)^**^ |
| GlaucomaNet | **0.969 (0.005)** | **0.983 (0.007)** | **0.929 (0.020)** | **0.992 (0.003)** | **0.955 (0.008)** | **0.997 (0.001)** |

^†^ The model was trained on 10,928 images with 4,528 having POAG. Reprinted from Li L, Xu M, Liu H, et al. A large-scale database and a CNN model for attention-based glaucoma detection. IEEE transactions on medical imaging 2019;39(2):413-24. Reprinted with permission.
